# Limited Quantum Helium Transportation through Nano-channels by Quantum Fluctuation

**DOI:** 10.1038/srep28992

**Published:** 2016-07-01

**Authors:** Tomonori Ohba

**Affiliations:** 1 Graduate School of Science, Chiba University, 1-33 Yayoi, Inage, Chiba 263-8522, Japan

## Abstract

Helium at low temperatures has unique quantum properties such as superfluidity, which causes it to behave differently from a classical fluid. Despite our deep understanding of quantum mechanics, there are many open questions concerning the properties of quantum fluids in nanoscale systems. Herein, the quantum behavior of helium transportation through one-dimensional nanopores was evaluated by measuring the adsorption of quantum helium in the nanopores of single-walled carbon nanohorns and AlPO_4_-5 at 2–5 K. Quantum helium was transported unimpeded through nanopores larger than 0.7 nm in diameter, whereas quantum helium transportation was significantly restricted through 0.4-nm and 0.6-nm nanopores. Conversely, nitrogen molecules diffused through the 0.4-nm nanopores at 77 K. Therefore, quantum helium behaved as a fluid comprising atoms larger than 0.4–0.6 nm. This phenomenon was remarkable, considering that helium is the smallest existing element with a (classical) size of approximately 0.27 nm. This finding revealed the presence of significant quantum fluctuations. Quantum fluctuation determined the behaviors of quantum flux and is essential to understanding unique quantum behaviors in nanoscale systems.

Molecules adsorbed in nanometer-scale pores (nanopores) have attracted much attention because of their unique properties, which exist as a result of high adsorption potentials and restricted spaces. Such unique properties are desirable in various applications of storage, separation, and selective reactions^1^,^2^,^3^,^4^,^5^,^6^. For instance, selective carbon dioxide removal is possible by using nanoporous materials such as nanoporous carbons, zeolites, or metal organic frameworks^7^,^8^,^9^,^10^,^11^,^12^. Additionally, the high density storage of methane and hydrogen as clean fuel gases by nanoporous materials has been reported at high pressures^13^,^14^,^15^,^16^. The adsorption properties of such typical molecules can be understood by using molecular simulation employing classical models^17^,^18^,^19^. However, quantum effects additionally need to be considered for light molecular adsorption at low temperatures. Quantum fluctuation at zero temperature induced the increase of effective hard sphere radii, providing large density fluctuations^20^. Large phase fluctuation in Bose-Einstein condensates was also observed nearly 0 K^21^. Quantum fluctuations thus became significant than thermal fluctuation at very low temperature^22^. However, the effect of quantum fluctuations of hydrogen and water molecules were also observed even above 20 K^23^,^24^,^25^. Here, the increase of effective radii of a helium atom by quantum effect above 0 K is also named as quantum fluctuation.

Hydrogen isotopes have been separated according to their quantum effects in carbon nanotubes at 20 K^23^. Moreover, as demonstrated, quantum effects are efficient toward the selective adsorption of hydrogen isotopes even at 77 K^26^,^27^. Nguyen *et al*. also proposed that quantum effects are remarkable for the diffusion of hydrogen isotopes in carbon nanopores at temperatures below 80 K^28^. Thus, determining the quantum effects of adsorbed molecules is essential to better understand molecular properties in nanopores at low temperatures. Those quantum effects could associate with quantum fluctuation of molecules. Helium, which is one of the lightest particles, spherical, and not solidified at any temperatures at ambient pressure, shows unique quantum phenomena such as superfluidity and λ-transition^29^,^30^,^31^,^32^. Thus, performing helium adsorption studies is suitable for evaluating quantum molecular properties in nanopores. Helium is also known to exhibit superfluidity in low-dimensional nanopores^33^, with the superfluid transitions in such nanopores being strongly related to the nanopore size, connectivity, and dynamical suppression of the quantum phase^34^,^35^,^36^,^37^,^38^. Quantum dispersion should correspond to the configuration space of the helium atoms; thus, the behaviors of quantum dispersion are considerably influenced by space limitation. However, our understanding of the quantum mechanics of such systems, especially quantum dispersion associated with the uncertainty principle, is still limited. In this paper, the adsorption of quantum helium in one-dimensional channels of carbon and zeolite nanopores was observed by experimental adsorption isotherms of helium at 2–5 K associated with simulated helium adsorptions. The penetration of quantum helium through various nanopores was assessed to show the occurrence of quantum fluctuations in nanopores.

## Results and Discussion

**Figure 1:**
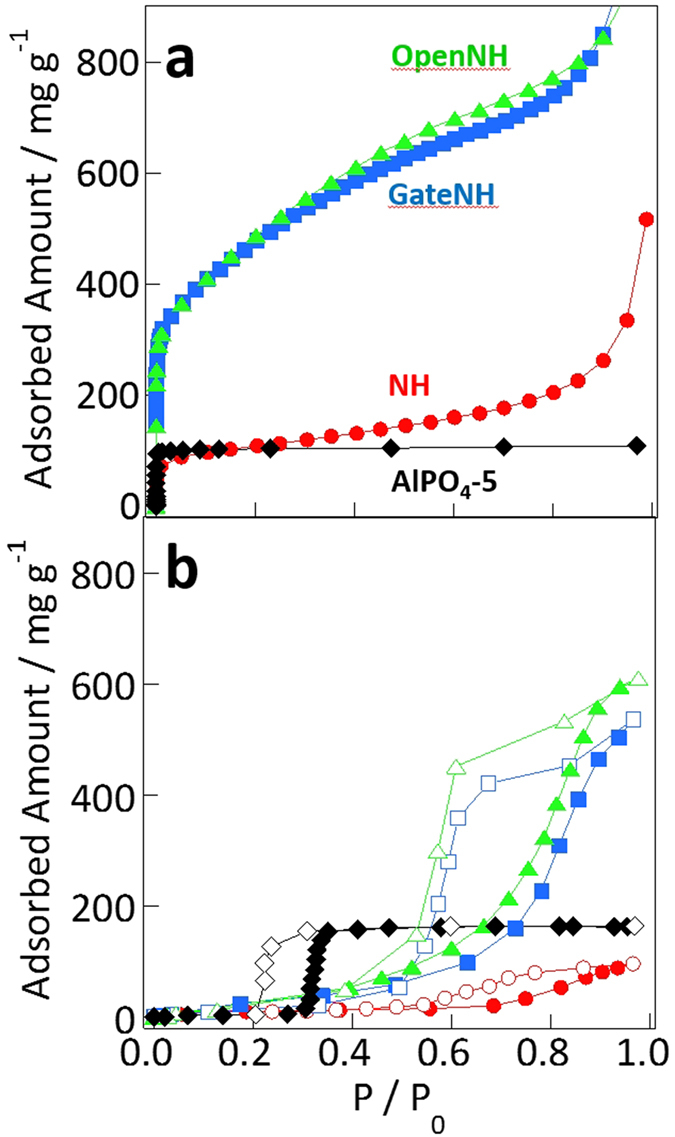
Structural evaluation of the studied nanoporous systems: (**a**) N_2_ adsorption isotherms measured at 77 K and (**b**) water vapor adsorption–desorption isotherms measured at 303 K. The curves with closed and open symbols represent the adsorption and desorption data, respectively.

The nanopore sizes and volumes were evaluated from the nitrogen and water vapor adsorption isotherms of Single-walled carbon nanohorns (NHs) and AlPO_4_-5 (Fig. 1). The specific surface areas and nanopore volumes (Table 1) were evaluated from Brunauer–Emmett–Teller and Dubinin–Radushkevich analyses of the nitrogen and water vapor adsorption isotherms^39^,^40^. The nanopore volumes of NH, GateNH (defined subsequently), OpenNH (defined subsequently), and AlPO_4_-5 evaluated from the nitrogen and water vapor adsorption isotherms were similar. The variations in the nanopore volume measurements were between 0 and 10%, suggesting the small size dependence of the adsorbed molecules in those nanopores. Here, the molecular sizes of N_2_ and water were approximately 0.4 and 0.3 nm, respectively. The average nanopore sizes shown in Table 1 were obtained from the literature^41^,^42^,^43^,^44^. The internal and interstitial nanopores of the NHs were cylindrical with diameters of 2.9 and 0.7 nm, as reported elsewhere^43^,^45^. AlPO_4_-5 featured uniform cylindrical nanopores of 0.7 nm in diameter, as determined from the crystal structure. Those pore sizes, evaluated from the nitrogen adsorption isotherms, were defined as surface-to-surface distances. The NHs with gates opening into the internal nanopores (referred to as GateNH) featured bottle neck-type cylindrical nanopores with diameters of 2.9 nm (bottle part), and diameter and thickness of 0.4 and 0.3 nm, respectively (neck part), prepared by partial oxidation of NH for 0.5 h in O_2_ atmosphere at 673 K^5^,^46^. NHs without neck regions (referred to as OpenNH) were prepared by partial oxidation of NH for 9.0 h in O_2_ atmosphere at 67 K for comparison with NH and GateNH^46^,^47^. Nitrogen and water molecules were fully adsorbed into the internal nanopores through the 0.4-nm-diameter neck regions. Thus, all evaluated nanopores were considered to be permeable to helium atoms because the classical atomic size of helium is 0.27 nm^48^.

**Figure 2:**
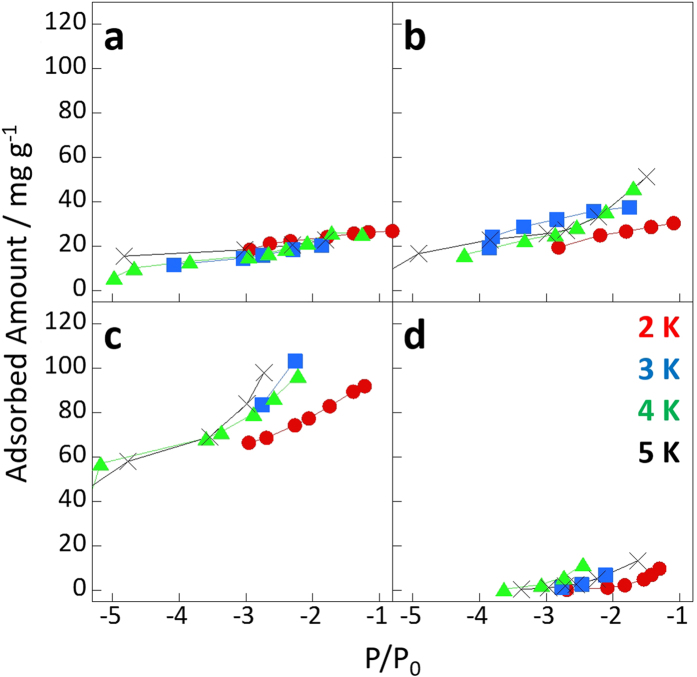
Helium adsorption isotherms of NH (**a**), GateNH (**b**), OpenNH (**c**), and AlPO_4_-5 (**d**) measured at 2–5 K.

**Figure 3:**
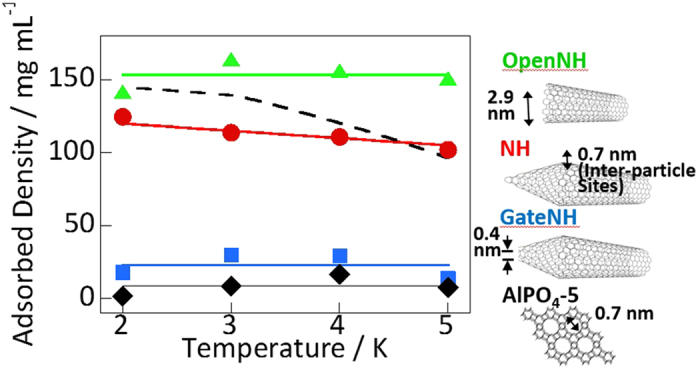
Variations in the helium density in low-dimensional nanopores of different sizes as a function of temperature. The bulk density is also shown for comparison (black broken curve). Structural images showing the effective nanopore sizes are shown on the right of the graph.

To examine the accessibility of the nanopores to helium, the helium adsorption isotherms of NHs and AlPO_4_-5 were measured at 2–5 K (Fig. 2). The amount of helium adsorbed into OpenNH was three times larger than that adsorbed into either NHs or GateNH; the amounts adsorbed on NH and GateNH were similar. Thus, helium could only be adsorbed in the interstitial nanopores. Furthermore, very small amounts of helium were adsorbed in the pores of AlPO_4_-5. Figure 3 shows the helium density in the nanopores obtained from the adsorbed amounts of helium at *P/P*_0_ = 0.001 in Fig. 2 and nanopore volumes evaluated from the nitrogen and water vapor adsorption isotherms (Fig. 1). The helium densities in the nanopores of NH and OpenNH were similar to the bulk densities, thereby suggesting the unlikely occurrence of pore blocking and nanopore volumes evaluated from the nitrogen and water vapor adsorption as mentioned above. However, the measured densities were independent or less dependent on temperature than the bulk densities. The helium densities in the nanopores of OpenNH were higher than those of bulk liquid helium, whereas those in NHs were much lower than those of bulk liquid helium. The smaller temperature dependence with the relatively high density was also observed in the narrow slit nanopores in a previous study, and this observation may be a result of a supercondensed state^49^. In contrast, the helium density in the AlPO_4_-5 nanopores was extremely low, indicating that the helium atoms could not penetrate the 0.7-nm-diameter nanopores despite having an atomic size of 0.27 nm (determined from the classical model). Despite having the same nanopore diameters, helium adsorption was rarely observed in AlPO_4_-5 and well observed in NH. These results indicated that NH, which had a nanopore size distribution, promoted helium adsorption owing to the presence of nanopores larger than 0.7 nm. In contrast, the crystalline nanopores of 0.7 nm in diameter in AlPO_4_-5 forbade helium adsorption. The 0.4-nm-diameter gates of GateNH also forbade helium penetrating into its internal nanopores. The helium densities in GateNH were considerably smaller than those in the other NHs as mentioned above. This result indicated that helium could be adsorbed in the interstitial nanopores of GateNH, whereas helium could not be adsorbed in the internal nanopores. Thus, helium atoms could not penetrate via the neck regions of GateNH (0.4-nm gates) and the nanopores of AlPO_4_-5 (0.7-nm nanopores).

**Figure 4:**
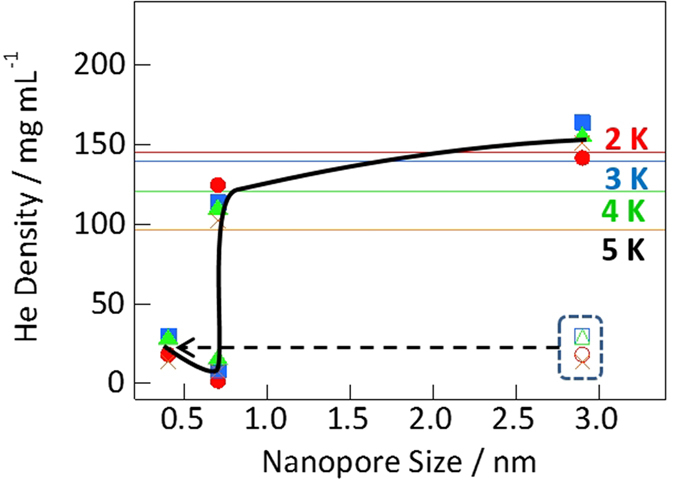
Dependence of experimental helium density on the nanopore size at 2 (●), 3 (■), 4 (▲), and 5 K (×). Solid lines represent the bulk helium densities.

The quantum dynamic properties of helium confined in nanopores were clearly observed by evaluating the helium densities as a function of nanopore size at 2–5 K (Fig. 4). The helium density in nanopores smaller than 0.7 nm was significantly low, whereas that in nanopores larger than 0.7 nm was similar to the bulk density. The preceding study reporting on the helium density in slit nanopores of 0.7–1.1 nm suggested that helium could be adequately adsorbed in those nanopores^49^. The transportation of helium atoms was surprisingly impeded through nanopores of 0.7 nm in diameter in AlPO_4_-5 and 0.4 nm in diameter in GateNH despite these nanopores being larger than the atomic size of helium. NH gates inhibited the penetration of helium despite having a gate length of 0.3 nm and into the large nanopores of 2.9 nm in diameter because typical pore blocking was observed in long narrow nanopores only. Furthermore, the relatively poor helium penetration through the 0.7-nm nanopores of AlPO_4_-5 could not be expected owing to typical pore blocking effects as the nanopores (0.7 nm in diameter) were more than twice as large as the atomic size of helium (0.27 nm). Thus, nanopores narrower than 0.7 nm in diameter inhibited the adsorption of helium atoms by blocking helium atoms. In the bottle neck-type NH nanopores, with bottle regions of 2.9 nm in diameter and neck regions of 0.4 nm in diameter, helium penetration via the 0.4-nm nanopores occurred to a certain extent rather than the 0.7-nm nanopores in AlPO_4_-5. Despite the nanopores being narrower in the neck regions of GateNH than the AlPO_4_-5 nanopores, the helium density in the former was higher than that in the latter. Thus, some helium atoms could penetrate the thin neck regions of GateNH owing to quantum tunneling beyond a potential barrier. The likelihood of such quantum tunneling through the nanopores of graphene sheets was recently demonstrated theoretically^50^,^51^.

**Figure 5:**
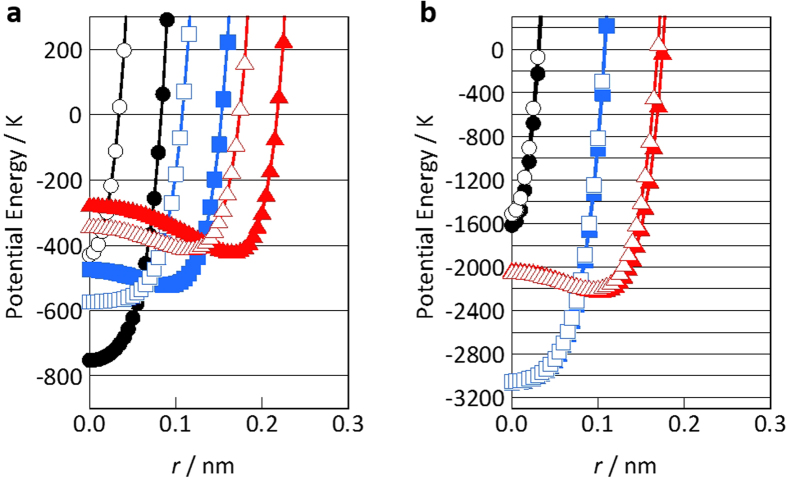
Potential profiles of a helium atom (**a**) and a nitrogen molecule (**b**) confined in the nanopores of CNTs of diameters of 0.68 (●), 0.81 (■), and 0.95 nm (▲). *r* is the distance from the CNT centers. Opened and filled symbols represent potential profiles in quantum and classical models, respectively. Here the zero potential energy position is at infinite distance from a CNT.

For a better understanding of the above experimental results path-integral canonical ensemble molecular dynamics simulations of helium adsorption have been performed. The potential energies for the adsorption of helium and nitrogen in the carbon nanotubes (CNTs) are plotted as a function of the distance from the carbon nanotube center. Here the classical potentials and quantum effective potentials in Fig. 5 were calculated using the Lennard-Jones potential and Feynman-Hibbs effective potential at 4 K, respectively, although the Feynman-Hibbs effective potential of helium is a rough quantum approximation^52^. Difference of helium potential profiles in quantum and classical models at 4 K was significant, whereas those of nitrogen at 77 K were indistinguishable from each other. Thus, helium adsorbed in CNTs at 4 K have to be treated as a quantum particle, although nitrogen could be considered as a classical molecule. Higher potential depths were observed as the pore diameters became narrower and those potential minima were along the CNT walls. Deeper potential wells of helium in the narrower diameters were explained by the larger number of pairwise interactions with the contacted carbon atoms. Nitrogen has stronger interaction potentials with a CNT wall than helium interaction potentials, as shown in Fig. 5. However, the nitrogen adsorption potential in the CNT nanopore of 0.68 nm diameter in Fig. 5b was shallower than the others, because the molecular size was slightly larger than the effective CNT diameter. The effective CNT diameters, which corresponded to the experimentally obtained pore sizes, were calculated from the sum of the distances at the potential energy of zero and collision diameter. The effective CNT diameters corresponding to 0.68, 0.81, and 0.95 nm were approximately 0.4, 0.6, and 0.8 nm for helium, respectively.

**Figure 6:**
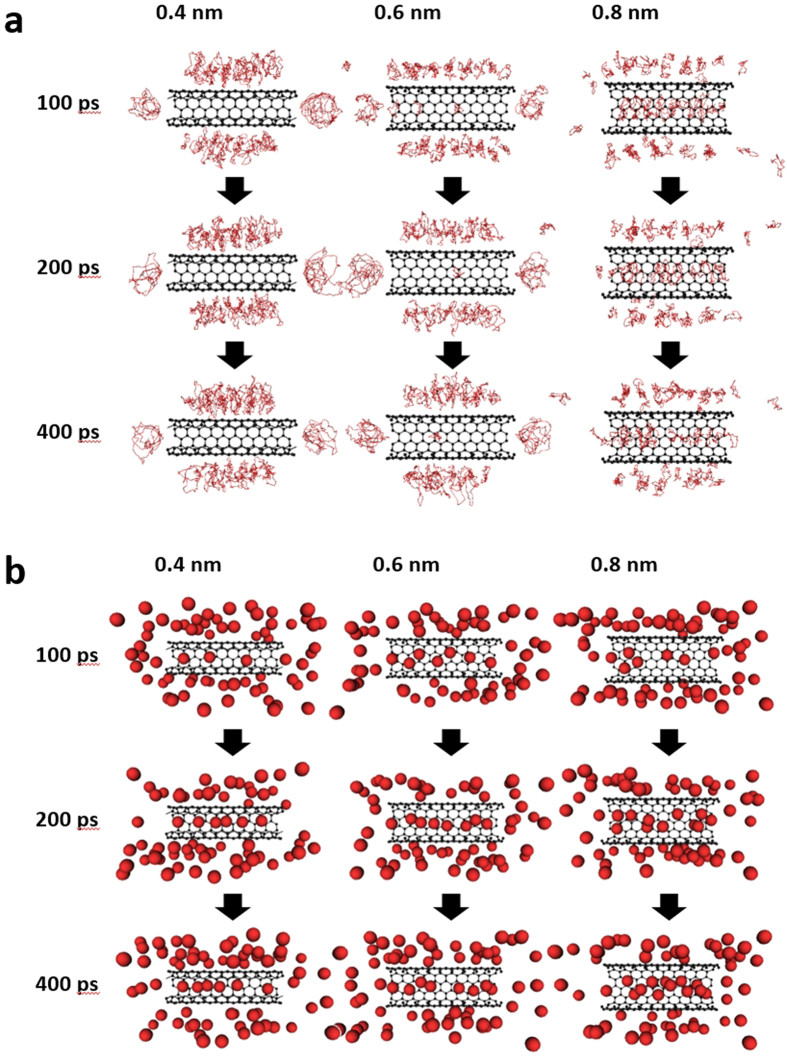
Snapshots of (**a**) quantum helium and (**b**) classical helium in the vicinity of CNTs with effective diameters of 0.4, 0.6, and 0.8 nm. The red curves, red spheres, and black represent the quantum helium atoms, classical helium atoms, and carbon atoms of the CNTs, respectively.

**Figure 7:**
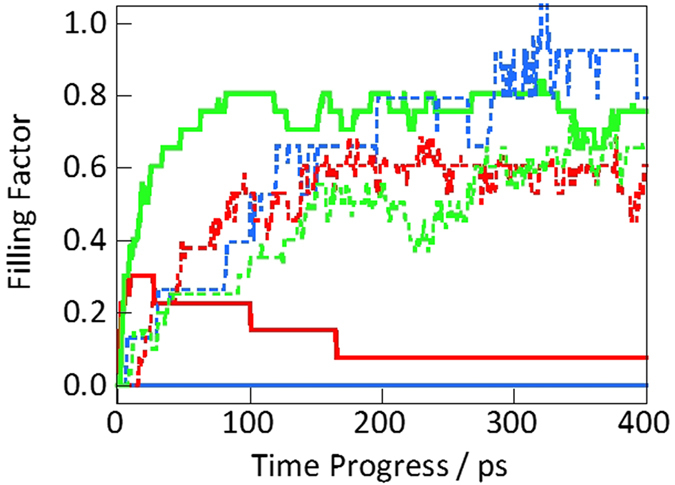
Helium penetration through the internal nanopores of CNTs evaluated by measuring the filling factor in CNTs of effective diameters of 0.4 (blue curve), 0.6 (red curve), and 0.8 nm (green curve). Helium penetration through the 0.3-nm gate of a CNT is represented by the black curve. The solid and dashed curves represent the helium penetration data according to the quantum and classical models, respectively.

**Figure 8:**
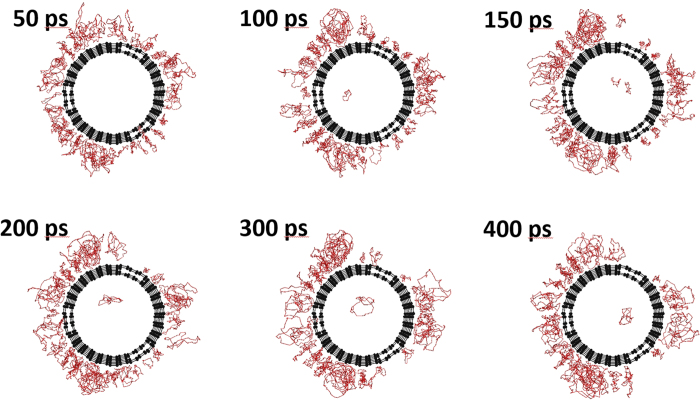
Snapshots of quantum helium penetration into a CNT via 0.3-nm gates. The red curves and black spheres represent the polymer rings of quantum helium atoms and the carbon atoms of a CNT, respectively.

**Figure 9:**
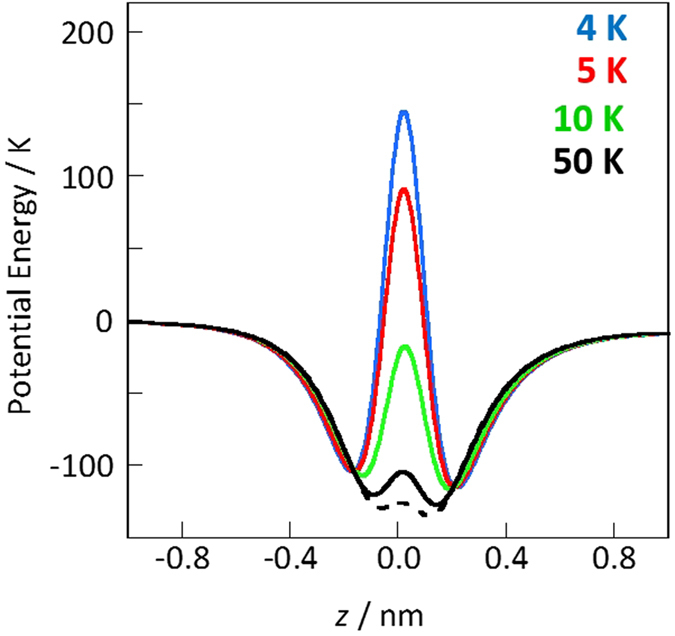
Potential profiles of a quantum helium atom (solid curves) via the 0.3-nm graphene gate at 4, 5, 10, and 50 K. The potential profile of a classical helium atom is shown for comparison (dashed curve). The graphene gate was positioned at *z* = 0, and plus and minus on the abscissa represent in the internal and external sites of a CNT.

Harmonic oscillators could describe the quantum fluctuations on the ground-state energy of a helium atom as well as Feynman-Hibbs effective potential^53^. In this study, the path-integral molecular dynamics simulations were used for evaluating the quantum fluctuation and penetration of helium through those nanopores. A path-integral calculation assumes ring polymers of a particle for description of quantum fluctuations, as explained in the Methods. Figure 6 shows snapshots of the position of quantum and classical helium within the vicinity of a CNT obtained via molecular dynamics simulations. Here, half of a CNT and helium atoms nearby the CNT were depicted for visibility. The quantum helium atoms in Fig. 6a were adequately adsorbed in the 0.8-nm nanopores. In contrast, quantum helium atoms could not penetrate the CNT nanopore of 0.4 nm in diameter until after 400 ps. Few helium atoms could enter the 0.6-nm nanopore of the CNT. In contrast, classical helium atoms could be adsorbed in all of the inner regions of the nanopores (Fig. 6b). The potential profiles along CNT axis in Fig. S1 also suggested that a helium atom is stabilized in the inner part of CNTs without any energy barriers. The adsorptions of nitrogen in CNTs at 77 K were also observed in all of the inner regions of the nanopors (Fig. S2), but the CNT nanopore of 0.4 nm rarely adsorb nitrogen due to the shallower adsorption potential (Fig. 5b). The different adsorption properties of helium and nitrogen were thus observed owing to mainly different molecular size. The atomic numbers of helium that penetrated the CNTs in the quantum and classical models as a function of time, as shown in Fig. 7, were calculated from the snapshots in Fig. 6. Here, the filling factor in Fig. 7 is defined as the adsorbed helium densities in the internal nanopores of CNTs divided by the bulk liquid density (120 mg mL^−1^) at 4 K. The filling factors obtained from the classical models were approximately 0.6–0.9 at 400 ps. The quantum helium atoms in the 0.7-nm CNTs system entered the internal regions of the nanopores more quickly than the classical helium atoms regardless of the type of nanopores. Quantum helium atoms were slightly adsorbed in the 0.6-nm CNTs system and could not be adsorbed in the 0.4-nm CNTs system, although classical helium could be adsorbed in all CNTs systems as mentioned above. The significant restriction of quantum helium adsorption in the 0.4- and 0.6-nm CNTs systems was attributed to quantum fluctuations of the helium atoms at 4 K despite the large potential well depth in the classical model (Fig. 5a). This tendency was also observed in the quantum and classical potential models in Fig. 5a. In other words, the effective size of quantum helium atoms was larger owing to quantum fluctuation. The experimentally observed densities of 30 and 17 mg mL^−1^ in the 0.4- and 0.7-nm nanopores corresponded to filling factors of 0.25 and 0.14, respectively. Thus, the experimental adsorption density in the 0.7-nm nanopores approximately coincided with the simulated value of 0.2 for the 0.6-nm CNTs system. In contrast, the experimental density in the 0.4-nm nanopores was much higher than the simulated value of 0.0. Here, the nanopore length in the simulation was 2.5 nm, whereas that in the experiment was 0.34 nm, corresponding to the graphene thickness in the NH gates. To assess the penetration of helium through narrow and thin nanopores, molecular dynamics simulation of quantum helium penetration into the internal nanopores via the 0.3-nm graphene gates was conducted. Some helium atoms could penetrate the gates despite the nanopores being extremely narrow, as shown in Figs 7 and 8. The gate-size-dependence of penetration properties of various molecules were observed elsewhere^5^,^50^,^54^,^55^,^56^. Thus, graphene sheets with a width of 0.3 nm and thickness of 0.34 nm enabled quantum helium penetration when compared with the long CNTs with a width of 0.4 nm. Figure 9 shows the potential profiles of a quantum helium atom penetrating through the 0.3-nm graphene gate. The quantum effective potentials were calculated using Feynman-Hibbs effective potential^52^. The energy barriers were negligible at 50 K as well as in the classical model. However, the energy barriers were increased with decreasing temperature, caused by the increase of effective molecular size of quantum helium. The energy barriers of quantum helium via the graphene gates and size-dependence of energy barriers were also observed in the preceding study^51^. The energy barrier, 250 K at 4 K was considerably higher than the kinetic energy. However, some helium atoms penetrated through the gates despite the high energy barrier at the gate, as shown in Fig. 8. Thus, the extremely narrow potential barrier via the gates of NH instigated helium penetration through the 0.4-nm nanopores by quantum tunneling.

The present study illustrates the unique quantum behavior of helium confined in 0.4–2.9-nm-diameter nanopores, including the loss of its temperature-dependent properties. The presence of quantum fluctuations prevented helium from penetrating narrow nanopores of less than 0.7 nm in size. Although those pores were larger relative to the atomic size of helium, penetration through the nanopores was restricted by quantum fluctuations in the path-integral molecular dynamics simulations. The penetration barrier was only observed in the quantum simulations. Nevertheless, few helium atoms penetrated the 0.4-nm-diameter and 0.34-nm-thick nanopores, likely owing to quantum tunneling effects, when compared with the 0.7-nm nanopores. To the best of the author’s knowledge, this is the first experimental observation of quantum tunneling of helium through nanopores. The anomalous helium properties in nanopores by quantum fluctuation reported in this study represent an important contribution to our fundamental understanding of quantum science. Further studies on the penetration process of quantum helium in nanopores are necessary to better understand the dynamics of quantum fluctuation.

## Methods

### Experimental

NHs and AlPO_4_-5 were used as the nanoporous materials. The nanoporous structures were evaluated by α_S_-analysis of the corresponding nitrogen adsorption isotherms at 77 K and water vapor adsorption isotherms at 303 K after evacuation at 423 K below 10 mPa for more than 2 h using a volumetric apparatus (Autosorb-1, Quantachrome Instruments Co., Florida, USA). Three types of NHs were prepared by partial oxidation for opening the NH gates for subsequent evaluation of adsorption phenomena inside the nanopores. Helium adsorption isotherms were measured with an in-house-built volumetric adsorption apparatus attached to a superconducting quantum interference device (MPMS XL, Quantum Design Inc., California, USA) to control the temperature. Prior to the measurements, the nanoporous materials were evacuated at 373 K below 10 mPa for more than 2 h in the device after drying in air. Helium was then gradually introduced into the sample cell.

### Simulation Procedure

Path-integral canonical ensemble molecular dynamics simulations of helium adsorption were performed to observe the penetration and adsorption of quantum helium in carbon nanopores of different sizes of 0.68, 0.81, and 0.95 nm (carbon-center-to-carbon-center diameter) as well as classical helium and nitrogen adsorptions. The simulations employed the leapfrog Verlet integration algorithm in the *NVT* ensemble with coupling to a thermal bath held at approximately 4 K. For the path-integral calculations, a helium atom was replaced by a classical ring polymer composed of polymer beads. Each polymer bead was connected to adjacent polymer beads in the same ring by harmonic springs. In this manner, a helium atom was considered as a quantum particle and the path-integral partition function of helium is described by ring polymers, as shown in eq. 1.


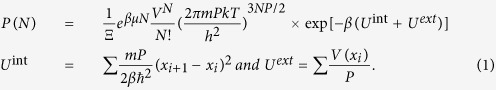


where *V, N, m, P, k, T*, and *h* denote the volume of the unit cell, number of helium, mass of an atom, polymer beads number, Boltzmann constant, temperature, and Planck constant, respectively. Inter-beads and interatomic interactions were described by *U *^int^ and *U *^ext^, respectively. Inter-beads and interatomic potential models were in the form of harmonic potential and Lennard–Jones potential *V*(*x*_*i*_), respectively. Those potential models are commonly used to describe quantum systems, as reported elsewhere^32^,^57^,^58^,^59^,^60^,^61^. The path-integral partition function was also adopted for the carbon atoms.

The potential parameters of helium and carbon atoms used in this study are as follows: the collision diameter and potential well depth of a helium atom are 0.256 nm and 10.2 K, respectively; those of a nitrogen molecule are 0.3416 nm and 104.2 K; those of a carbon atom are 0.34 nm and 28.0 K, respectively^62^,^63^,^64^,^65^. The Lorentz–Berthelot mixing rules were applied in the potential calculations between the helium and carbon atoms, and three-dimensional periodic boundary conditions were applied. The number of helium and carbon polymer beads used in the simulation was 30. The classical helium behaviors were also determined using one bead only. The unit cell size of 4.0 × 2.0 × 2.0 nm^3^ was chosen for all the CNTs. CNTs of 2.46 nm in length were aligned to the *x*-axis. Bulk spaces were defined as follows: *x* > + 1.5 nm or <−1.5 nm. Hundred helium atoms, which roughly correspond to half of the liquid density in bulk spaces at 4 K, were arranged in the external nanopores of the CNTs. Gate penetration into the graphene walls was assessed using a CNT with 0.3-nm gates by removal of 12 carbon atoms. Hundred helium atoms were positioned in the external spaces of a CNT in the unit cell of 1.7 × 3.0 × 3.0 nm^3^, and the amount of helium penetrating the internal nanopores was determined. The simulations were run for 400 ps using an integration time step of 0.2 fs.

## Additional Information

**How to cite this article**: Ohba, T. Limited Quantum Helium Transportation through Nano-channels by Quantum Fluctuation. *Sci. Rep.* **6**, 28992; doi: 10.1038/srep28992 (2016).

## Supplementary Material

Supplementary Information

## Figures and Tables

**Table 1:** Nanopore structures of NH, GateNH, OpenNH, and AlPO_4_-5 evaluated from Dubinin–Radushkevich analyses of N_2_ and water vapor adsorption isotherms.

	Specific Surface Area/m^2^ g^−1^	Nanopore Volume/mL g^−1^		Nanopore Diameter/nm
		From N_2_	From Water	
NH	280	0.13	0.13	0.7 (<1.3 nm)
GateNH	1300	0.51	0.48	2.9 (<4 nm)
OpenNH	1350	0.53	0.56	2.9 (<4 nm)
AlPO_4_-5	270	0.14	0.16	0.7
