# Supplementary material for: Limited Quantum Helium Transportation through Nano-channels by Quantum Fluctuation
Source: Sci Rep. 2016 Jul 1;6:28992. doi: 10.1038/srep28992 (PMC4929499; doi:10.1038/srep28992)
Supplement: Supplementary Information [file srep28992-s1.pdf]

[Supporting Information]

Limited Quantum Helium Transportation through Nano-channels by Quantum Fluctuation

Tomonori Ohba

† Graduate School of Science, Chiba University, 1-33 Yayoi, Inage, Chiba 263-8522, Japan

*To whom correspondence should be addressed. E-mail: ohba@chiba-u.jp

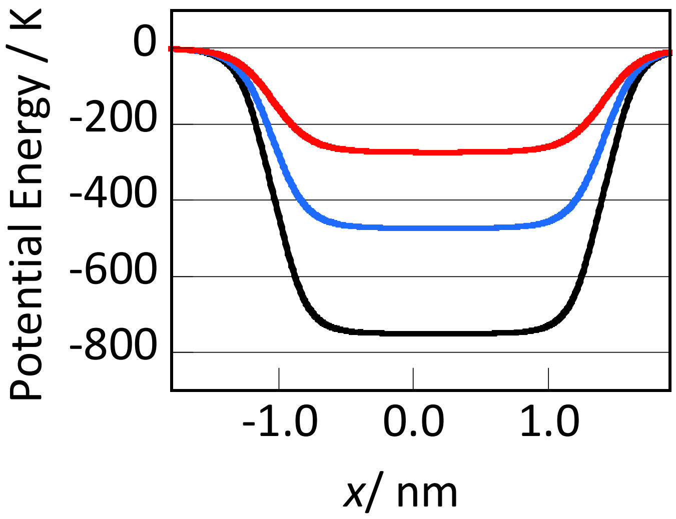

Figure S1 Potential profiles of a classical helium atom as function of CNT axis for 2.46 nm CNTs of diameters of 0.68 (black curve), 0.81 (blue curve), and 0.95 nm (red curve).

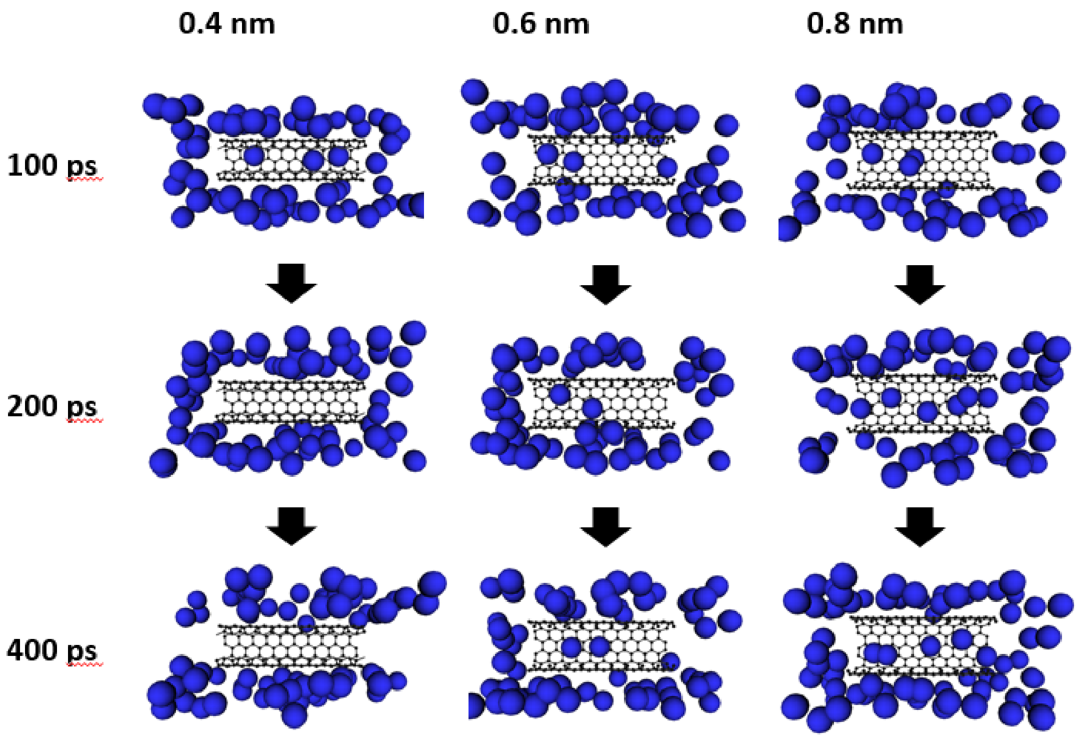

Figure S2 Snapshots of nitrogen in the vicinity of CNTs with effective diameters of 0.4, 0.6, and 0.8 nm. The blue spheres and black honeycomb structures represent the nitrogen molecules and carbon atoms of the CNTs, respectively.
